# Levels of bioactive endogenous lipids and health-related quality of life in Chronic Idiopathic Axonal Polyneuropathy

**DOI:** 10.48101/ujms.v127.8577

**Published:** 2022-05-30

**Authors:** Jonas Lind, Niclas Stensson, Björn Gerdle, Nazdar Ghafouri

**Affiliations:** a Department of Neurology, Internal Medicine, County Hospital Ryhov, Jönköping, Sweden; b Division of Neurobiology, Department of Biomedical and Clinical Sciences, Linköping University, Linköping, Sweden; c Pain and Rehabilitation Centre, and Department of Health, Medicine and Caring Sciences, Linköping University, Linköping, Sweden

**Keywords:** Endocannabinoids, *N*-Acylethanolamines, pain, polyneuropathy, neuralgia, neuropathic pain, plasma biomarkers

## Abstract

**Background:**

Although neuropathic pain is a significant problem in polyneuropathy, the underlying molecular mechanisms are poorly understood. The endogenous bioactive lipids 2-arachidonoyl-glycerol (2-AG), oleoylethanolamide (OEA), palmitoylethanolamide (PEA), and stearoylethanolamide (SEA) are known to influence pain and inflammation in the peripheral nervous system. The aim of this study was to explore the plasma levels of endocannabinoids and related lipids and health-related quality of life in patients with polyneuropathy with and without pain.

**Methods:**

Patients (*n* = 48) with Chronic Idiopathic Axonal Neuropathy were included. Clinical data were retrieved from medical files. All patients filled out the SF-36 and EQ-5D questionnaires. In addition, blood samples were analyzed for 2-AG, OEA, PEA, and SEA.

**Results:**

Neuropathic pain was reported in 21 of the patients. There were significantly lower levels of 2-AG in patients with neuropathic pain (*P* = 0.03), but there were no significant differences in OEA (*P* = 0.61), PEA (*P* = 0.95), or SEA (*P* = 0.97) levels. The patients reporting pain in the hands had significantly lower SEA levels, 10.0 versus 15.0 (*P* = 0.03). The levels of 2-AG were significantly higher among patients reporting paresthesia in their feet (80.1 vs. 56.3; *P* = 0.02). Levels of PEA, SEA, and 2-AG were decreased in patients with loss of vibration. PEA and SEA were decreased in patients with loss of pain and temperature, and SEA decreased in patients with loss of sense of touch. However, the differences in the levels of bioactive endogenous lipids were not statistically significant when corrected for multiple comparisons.

**Conclusion:**

Alterations of 2-AG levels between polyneuropathy patients with and without neurogenic pain indicate that it could play an essential role. Further studies are warranted.

## Introduction

Neuropathies are common peripheral nervous system disorders affecting at least 4% of the general middle-aged and elderly population (1). The clinical features vary enormously, and patients may present various complaints, including muscle weakness and wasting, paresthesia, sensory impairment, and severe neuropathic pain (burning, lancinating, shooting, or tingling), seriously compromising their quality of life (2). The diagnostic evaluation involves electrophysiological testing, including nerve conduction studies, histopathologic analysis of nerve tissue, serum laboratory testing, and sometimes autonomic testing and analysis of the cerebrospinal fluid (3). About 10–20% of the patients with polyneuropathy have a Chronic Idiopathic Axonal Polyneuropathy (CIAP), which requires that no etiological cause is found after extensive assessment. We have previously investigated the clinical and neurophysiological characteristics of patients with CIAP (4), and the typical symptoms are weakness in distal muscles, peripheral sensory impairment, and sometimes additional neurogenic pain. Pain in both polyneuropathy in general and CIAP increases the adverse effects on the daily activities and quality of life (2, 5).

Neuropathic pain is defined by the International Association for the Study of Pain (IASP) as ‘pain caused by a lesion or disease of the somatosensory nervous system’ (6). Since there are no pain biomarkers, neuropathic pain is identified based on clinical criteria. However, the physical examination can only provide supporting evidence for a neurological lesion or disorder that could truly be the cause of pain (7).

The molecular mechanisms behind the pain mechanisms in polyneuropathy are poorly understood. The ‘gate control theory’ of pain has, since its formulation in 1965, postulated that neuronal circuits in the central nervous system (CNS) regulate nociceptive signals arising in the periphery of the body (8). The descending systems to control pain appear to have three functionally interrelated neurotransmitter mechanisms, the noradrenergic, the serotonergic, and the opioid systems (9). However, current understanding indicates that nociceptive signals may undergo a dynamic filtering process in the periphery before reaching the spinal cord (10).

Endogenous bioactive lipid mediators modulate the immune system (11) and influence pain and inflammation in the peripheral nervous system (10). These lipids include several subgroups like endocannabinoids (ECs) and endogenous peroxisome proliferator-activated receptor type-α (PPARα) agonists. For example, 2-arachidonoyl-glycerol (2-AG) is a full agonist at Cannabinoid Receptor 1 (CB1) and Cannabinoid Receptor 2 (CB2) and is involved in the descending modulation of pain during acute stress (12, 13), and 2-AG may also contribute to CB1 receptor tone (14). In cisplatin-induced polyneuropathy in both rats and mice, levels of 2-AG were decreased in the paw skin and increased in dorsal root ganglions (14), indicating that 2-AG might be of interest for further studies in neuropathies.

Naturally occurring N-acyl ethanolamines have been widely studied in the context of neuroinflammation and neuropathic pain in recent years (15). Neurons generate oleoylethanolamide (OEA) and palmitoylethanolamide (PEA) in the dorsal roots, skin cells, and many other cell types, even in the absence of external stimuli (10) and activate PPAR-α (16, 17). PPARs are nuclear receptors modulating immune and inflammatory reactions (18). OEA is associated with analgesic properties that may occur independently of PPAR-α activation and induce visceral pain (19). In addition, both PEA and OEA may modulate peripheral nociceptors’ excitability (10, 20). Stearoylethanolamide (SEA) has been proposed to activate PPAR-α (21) and to generate anti-inflammatory activity (22).

Chronic widespread pain (CWP) is often associated with fibromyalgia syndrome. Central alterations in CWP may be driven by peripheral alterations in muscles (23, 24) and/or small fibers (A-delta and C-fiber) (25, 26). Significantly higher OEA and PEA plasma levels were found in CWP (27), contrasting with rheumatoid arthritis, where lower OEA and PEA levels in synovial fluid were found (28).

There is a lack of knowledge about bioactive endogenous lipids in CIAP, and we hypothesize that they play an essential role in the regulation of pain. Hence, the aim of this study was to investigate the plasma levels of ECs and related lipids (2-AG, OEA, PEA, and SEA) and health-related quality of life in patients with polyneuropathy with and without neuropathic pain.

## Material and methods

This study’s subjects have previously been included in studies of clinical data and exposures in polyneuropathy (2, 4, 29–32). Outpatients (*n* = 255) were screened at the neurology departments at Linköping University Hospital and Ryhov Hospital in Jönköping, Sweden. Inclusion criteria were aged 40–79 years at diagnosis of CIAP. The patients were interviewed and examined at diagnosis according to clinical routine. We defined polyneuropathy as at least one typical polyneuropathy symptom and at least one of the clinical findings (distal deficit of sensation, reduced distal muscle strength, and impaired or lost deep tendon reflexes). The following laboratory investigations had to be normal to consider it as CIAP: hemoglobin, serum glucose, cobalamin, folate, and thyroid function.

Nerve conduction studies were not required, but they had to show axonal or combined axonal and demyelinating findings if performed. Patients with dominantly demyelinating neurophysiological findings alone or conduction block were excluded. Patients under the age of 40 were not included to avoid genetic or toxic polyneuropathies not yet diagnosed. Forty-eight patients fulfilled the criteria and gave consent to participate in this study. Blood samples and questionnaires were obtained after the written informed consent. Neuropathic pain was defined according to IASP (6). The pain was classified as neuropathic or not based on information in the medical files.

### Ethical approval

The regional ethical committee in Linköping approved the study (Dnr 99353 and Dnr 01-443), and the patients included gave their written informed consent.

### Data collection

The following data were retrieved from medical files: age, sex, year of first symptom and year of diagnosis, reported symptoms, clinical findings at the first visit, and neurophysiological findings. The severity of polyneuropathy was based on Perinea’s classification (33): ‘1 = minor motor and/or sensory symptoms without functional deficit; 2 = minor to moderate symptoms with a functional deficit, including slight ataxia; and 3 = severe symptoms with a functional deficit and at least some need for assistance’.

Of the 48 patients, 38 underwent neurophysiological testing to establish a diagnosis of axonal polyneuropathy and to exclude demyelinating polyneuropathy (see reference for methodology and neurophysiological criteria) (34).

### Mass spectrometry analysis

*N*-Acylethanolamines and 2-AG were analyzed in plasma samples using liquid chromatography tandem mass spectrometry (LC-MS/MS), based on a previously published method (35). Lipid extraction from plasma was based on a previously described protocol (36). Samples were thawed and vortexed, a 30 μL of a deuterated internal standard mix containing AEA-d4 (50 nM) and 2-AG-d5 (1,500 nM) was added to plasma and blank samples (300 μL), and a 1,200 μL acetonitrile (ACN) was added before vortexing and centrifugation (10,000 rpm, 5 min, 4°C) (Ann Arbor, MI, USA). The supernatant was added to 4.5 mL of millQ-H_2_O containing 0.133% trifluoroacetic acid (TFA). C8 Octyl solid-phase extraction columns (6 mL, 200 mg) (Biotage, Uppsala, Sweden) were used for further extraction. Before adding the sample to the column, they were activated with methanol (1 mL) and washed with millQ-H_2_O (1 mL), following a wash with 1.5 mL ACN (20% with 0.1% TFA). Elution was performed with 1.5 mL ACN (80% with 0.1% TFA). SpeedVacc then dried the eluate. The samples were reconstituted in 30 μL of LC mobile phase on analysis day, and 10 μL was the volume injected into the LC-MS/MS system.

An LC-MS/MS system containing a Thermo Scientific Accela AS autosampler and Accela 1250 pump coupled with a Thermo Scientific TSQ Quantum Access max triple quadrupole mass spectrometer with a HESI II probe as an ionization source was used. LC and MS/MS parameters were as in Ref. (35). The selected reaction monitoring (SRM) (m/z) transitions 326.3/62.4, 300.3/62.4, 328.3/62.4, and 379.3/287.3 were used for OEA, PEA, SEA, and 2-AG, respectively, and 352.3/66.4 and 384.3/287.3 for AEA-d4 and 2-AG-d5, respectively. Standard curves linearity measuring ranges were assessed from 5 to 1,000 nM for OEA, PEA, and SEA, and 25–5,000 nM for 2-AG. *R*^2^ ≥ 0.9 was the linearity for all analytes. All standards were purchased from Cayman Chemicals. The analytes were quantified using isotopic dilution according to the area ratio of their corresponding deuterated internal standard signal area. AEA-d4 corresponded for OEA, PEA, SEA, and 2-AG-d5 for 2-AG. Equal weighting and linear regression were applied. Peak integration and quantification were performed using the Xcalibur® (version 2.1, Thermo Scientific) software.

### Quality of life instruments

All patients filled out the SF-36 and EQ-5D questionnaires. Complete information about the methodology is described in earlier publications (2). Eight scales are derived in SF-36, including 1) physical functioning (PF), 2) role disruption due to physical difficulties (role disruption-physical; RP), 3) role disruption due to emotional difficulties (role disruption-emotional; RE), 4) social functioning (SF), 5) mental health (MH), 6) vitality (VT), 7) general health (GH), and 8) bodily pain (BP). In addition, two weighted summary scales were calculated: Mental Component Summary (MCS) and Physical Component Summary (PCS). Each scale has a scoring range from 0 to 100, with a higher score indicating better health. The Swedish version of the SF-36 was used with permission from the HRQL-group, Section for Healthcare Research Sahlgrenska University Hospital, Gothenburg, Sweden. EQ-5D consists of five items/dimensions: 1) mobility, 2) self-care, 3) usual activities (work, study, household, family, or leisure), 4) pain or discomfort, and 5) anxiety or depression. Each item has three answering alternatives: no problem, moderate problem, or extreme. The Swedish version of EQ-5D was used with permission from the EuroQol Group. An EQ-5D Index was calculated using the EQ-5D Index Value Calculator for Mac Version 1.0 developed by the EuroQol Group using Denmark’s value sets. EQ-5D also includes a visual analog scale (EQ-VAS; 0 denoting the worst imaginable health state and 100 the best imaginable health state).

### Statistics

Statistical analysis was performed using Statistica (version 13, Dell Inc., Tulsa, OK, USA). Data in text and figures are expressed as mean (standard deviation [SD]). For pairwise analysis of interval, a data *t*-test was performed. Because the SF-36 and the EQ-barometer scales are categorical and not normally distributed, the Kruskal–Wallis test was used, followed by Mann–Whitney U test. For EQ-5D, the Chi-2 test was used. The analysis was completed with Fischer’s exact test for groups with fewer than five respondents. Correlations were tested using Spearman’s rank correlation. Differences were considered significant if *P*-values were <0.05. For multiple comparisons, correction according to Bonferroni was performed.

## Results

**Figure 1:**
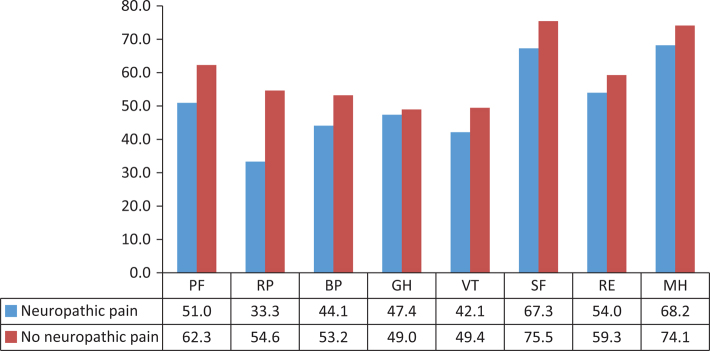
Short Form (36) Health Survey (SF-36) mean values for patients with or without neuropathic pain at diagnosis of Chronic Idiopathic Axonal Polyneuropathy (CIAP). No differences reached statistical significance.

Of the 48 patients included, 21 had symptoms and clinical findings of neuropathic pain. Patient characteristics are described in Table 1; there were no significant differences between the groups. There were no significant differences in SF-36 (Figure 1) or EQ-5D (Table 2) between patients with and without neuropathic pain.

**Table 1:** Background data are given as mean (±SD).

Group variables	Neuropathic pain (*n* = 21)	No neuropathic pain (*n* = 27)	Statistics *P*-value
Men (*n*)	14	19	
Women (*n*)	7	8	0.78
Age (years)	69.7 (9.1)	71.6 (7.5)	0.43
Age at diagnosis	61.0 (9.1)	64.2 (8.1)	0.20
Disease duration	12.3 (6.9)	11.3(6.0)	0.57

**Table 2:** EQ-5D: the five dimensions together with EQ-5D Index and EQ-VAS.

EQ-5D dimension			
Group	Neuropathic pain *n* = 21	No neuropathic pain *n* = 27	*P*
**Mobility**			
No problem	8	14	
Reporting problems	13	13	0.343
**Self-care**			
No problem	18	25	
Reporting problems	3	2	0.641
**Usual activity**			
No problem	10	18	
Reporting problems	11	9	0.184
**Pain/discomfort**			
No problem	2	5	
Reporting problems	19	22	0.445
**Anxiety/depression**			
No problem	9	11	
Reporting problems	12	16	0.883
**EQ-5D Index (mean ± SD)**	0.69 (±0.20)	0.68 (±0.24)	0.618
**EQ-5D-VAS (mean ± SD)**	57.2 (±19.5)	64.4 (±21.4)	0.140

Note: Note that the five items’ answering alternatives have been dichotomized (moderate problem and extreme problem taken together).

There were significantly lower levels of 2-AG in patients with neuropathic pain (*P* = 0.028), but there were no significant differences in OEA, PEA, or SEA levels (Table 3). The differences did not reach statistical significance when men and women were analyzed separately (data not shown). There were no differences in OEA, PEA, SEA, or 2-AG levels between men and women or smokers versus non-smokers. Levels of OEA and PEA were significantly correlated (Spearman Rho = 0.90, *P* < 0.001), as well as OEA and SEA (Spearman Rho = 0.70, *P* < 0.001), PEA and SEA (Spearman Rho = 0.76, *P* < 0.001), and SEA and AG (Spearman Rho = 0.34, *P* < 0.05). There was no correlation between 2-AG levels and levels of OEA and PEA. Age was affecting levels of OEA (Spearman Rho = 0.29, *P* < 0.05), but not PEA, SEA, or 2-AG.

**Table 3:** Concentrations of endocannabinoids in nM (mean (±SD) in patients with CIAP with or without neuropathic pain at diagnosis.

Substance	Neuropathic pain (*n* = 21)	No neuropathic pain (*n* = 27)	Statistics *P*-value
OEA	15.3 (8.4)	14.1 (7.1)	0.61
PEA	17.9 (8.7)	17.8 (8.8)	0.95
SEA	13.4 (7.5)	13.3 (7.9)	0.96
2-AG	56.6 (28.6)	78.9 (37.0)	0.03

OEA: oleoylethanolamide; PEA: palmitoylethanolamide; SEA: stearoylethanolamide; 2-AG: 2-arachidonoyl-glycerol.

The 16 patients reporting pain in the hands had significantly lower SEA levels, 10.0 (3.5) versus 15.0 (8.6) (*P* = 0.03). The levels of 2-AG were significantly higher among patients reporting paresthesia in their feet (80.1 vs. 56.3; *P* = 0.017). There were no other significant differences in levels of OEA, PEA, SEA, or 2-AG for any other symptoms from hands or feet.

Patients were examined for loss of the senses of vibration, pain, touch, loss of muscle strength, or loss of patella or heel tendon reflexes at diagnosis (Table 4). Levels of PEA, SEA, and 2-AG were decreased in patients with loss of vibration (Table 4). Lower levels of PEA were found in patients with loss of pain or temperature (Table 4). SEA was decreased in patients with loss of sense of touch (Table 4). The statistically significant differences in symptoms and clinical findings disappear when corrected for multiple comparisons.

**Table 4:** Mean (SD) concentrations in nM of OEA, PEA, SEA, and 2-AG in patients with normal function or loss of function at the neurological examination.

Clinical findings	OEA with loss of function	OEA normal	*P*	PEA with loss of function	PEA normal	*P*	SEA with loss of function	SEA normal	*P*	2-AG with loss of function	2-AG normal	*P*
Sense of vibration	14.1 (7.0)	21.4 (16.0)	0.118	17.2 (7.7)	27.8 (18.5)	0.044*	12.8 (6.8)	22.2 (16.4)	0.041*	66.7 (33.5)	113.1 (44.9)	0.028*
Sense of pain and temperature	13.7 (6.1)	16.4 (10.9)	0.309	15.7 (4.5)	22.0 (13.8)	0.032*	11.9 (5.9)	16.5 (10.9)	0.074	66.0 (36.7)	75.1 (35.2)	0.443
Sense of touch	13.9 (7.2)	16.5 (9.4)	0.320	16.8 (7.6)	20.6 (11.8)	0.209	11.7 (6.8)	17.1 (9.6)	0.040*	66.3 (37.9)	72.7 (27.9)	0.585
Muscle strength	13.1 (7.2)	17.1 (7.9)	0.072	16.8 (8.3)	19.5 (9.2)	0.288	12.4 (7.0)	14.8 (8.5)	0.304	64.3 (34.8)	76.6 (35.1)	0.238
Patellar tendon reflex	13.7 (7.3)	16.5 (8.2)	0.233	17.2 (8.2)	19.0 (9.6)	0.486	12.6 (6.9)	14.8 (8.9)	0.344	66.3 (34.4)	74.3 (36.6)	0.453
Heal tendon reflex	14.7 (8.2)	14.7 (5.5)	0.988	17.8 (9.2)	18.1 (6.7)	0.911	13.3 (8.3)	13.5 (3.6)	0.954	71.5 (36.7)	60.3 (27.8)	0.374

*OEA:* oleoylethanolamide; PEA: palmitoylethanolamide; SEA: stearoylethanolamide; 2-AG: 2-arachidonoyl-glycerol.

* Significant difference.

There were no significant correlations between SF-36 variables and levels of OEA, PEA, SEA, or 2-AG. PF, role physical, and PCS decreased with increasing clinical severity (Spearman Rho = −0.35, −0.33, and −0.30, respectively, *P* < 0.05), and other SF-36 variables OEA, PEA, and 2-AG were not correlated with clinical severity. Neither EQ-5D index nor EQ-VAS was correlated with OEA, PEA, SEA, or 2-AG levels. Neurophysiological severity was not correlated to any SF-36 or EQ-5D variables.

## Discussion

Painful neuropathy is a significant clinical problem with poorly known underlying mechanisms. Earlier reports on neuropathies and ECs have mainly been based on animal models. We explored the levels of endogenous lipids and found that the EC 2-AG levels in plasma are lower in patients with neuropathic pain than in patients without neuropathic pain in CIAP. Furthermore, levels of PEA, SEA, and 2-AG were decreased in patients with loss of sense of vibration, PEA was reduced in patients with loss of sense of pain and temperature, and SEA fell in patients with loss of sense of touch. Levels of OEA were associated with age.

Pain of any type is very common in patients with CIAP, affecting 85% of the patients (2). However, as the prevalence increases with age, pain is also common in the general population. In the same material, 56% of sex- and age-matched controls reported pain, emphasizing the importance of separating neuropathic pain from musculoskeletal pain or pain of other types.

2-AG is the major brain EC and a key regulator of neurotransmitter release involved in multiple (patho)physiological functions, such as emotion, cognition, energy balance, pain sensation, and neuroinflammation (37). 2-AG is produced upon demand from its precursors in the lipid membranes and released into the extracellular space, contrasting to classical neurotransmitters synthesized and stored in synaptic vessels (38).

Tonic release of endogenous PPAR-α agonists in healthy tissues, such as PEA and OEA, may help to set the threshold for nociception by regulating the baseline transcriptional activity of the NFκB complex and the opening of membrane ion channels in primary sensory afferents and nearby host-defense cells (10). A temporary interruption of OEA and PEA occurs after an injury. The biosynthesis caused by cell damage may disable the inhibitory effect exerted by these lipid messengers, letting the inflammation develop and nociceptive thresholds decrease. Simultaneously, the localized on-demand development of the ECs, anandamide, and 2-AG may moderate the exogenous and endogenous proalgesic agent effects by reducing nociceptor excitability and contrasting local pro-inflammatory signals. Finally, as the reaction to tissue injury moves toward its resolution stage, a wave of pain-relieving substances of oxidative polyunsaturated fatty acid metabolism, such as lipoxins and resolvins, may help to stabilize nociceptive reactions in the healing tissue (10).

Concentrating on endogenous regulators of neuroinflammation may be a feasible therapeutic approach for nervous system disorders by targeting non-neuronal cells. There are substantial CB1 levels in nociceptive and non-nociceptive sensory neurons of the dorsal root ganglions (39). Moreover, the capability of PEA to modulate shielding responses during inflammation and pain suggests that endogenous PEA may be an element of the multifaceted homeostatic system controlling the basal threshold of inflammation (‘modulator of immuno-neural homeostasis’) (11). In an animal model of neuropathy using chronic constriction injury, repetitive treatments with PEA reduced the presence of edema, and considerably thicker myelin sheaths, axonal diameters, and a higher number of fibers were found (40).

It has been suggested that OEA and PEA exert a tonic inhibitory control over the inflammatory and nociceptive responses (10). Our data indicate that they do not play an essential role in pain regulation in chronic non-inflammatory neuropathies. However, this should be interpreted with great caution, as plasma levels may not represent what is happening in the pathways regulating pain.

There has been great hope for cannabinoids as a treatment for neuropathic pain. However, data are still limited (41). In animal and human studies, cannabinoids have been found to alleviate allodynia and hyperalgesia associated with chronic inflammatory and neuropathic pain (42, 43). A rat model of chemotherapy-induced peripheral neuropathic pain found that a synthetic peripherally restricted drug potently suppresses painful neuropathy symptoms after local, systemic, and oral administration (44). Cannabinoids are regarded to be very effective in alleviating pain symptoms of chemotherapy-induced and other peripheral neuropathies. Still, they are not recommended in clinical practice due to the CNS-mediated adverse effects (44, 45).

### Strength and limitations

This is the first exploratory study of bioactive endogenous lipids in CIAP with clinical data and the patients’ perceived health-related quality of life. The differentiation between neuropathic and non-neuropathic pain was done from medical records. A prospective study using stringent criteria for neuropathic pain should be performed in the future. Our results are limited due to the low sample size and the lack of a healthy control group. The decreased levels of bioactive endogenous lipids in some symptoms and clinical findings should be interpreted with great caution as they were not statistically significant when corrected to multiple comparisons. However, our data mandate further studies.

## Conclusion

Neuropathic pain presents a significant burden to individuals and society. There is a need for a better understanding of underlying mechanisms. Earlier reports on neuropathies and ECs have mainly been based on animal models. Alteration of 2-AG levels between polyneuropathy patients with and without neurogenic pain indicates that 2-AG could play an essential role in neurogenic pain in patients with polyneuropathy.

## Disclosure statement

The authors report no conflicts of interest in this work.

## Funding

This study was supported by grants from the Swedish Research Council (2018-02470), County Council of Östergötland (Research-ALF; LIO-608021), and Futurum, Region Jönköping County. The sponsors had no role in study design, data collection, data analysis, data interpretation, writing of the report, or the decision to submit for publication. The authors had full access to all the data in the study and had final responsibility for the decision to submit it for publication.

## Data availability statement

The datasets generated and/or analyzed in this study are not publicly available as the Ethical Review Board has not approved these data’s public availability.

## Authors’ contributions

All authors made substantial contributions to conception and design, acquisition of data, or analysis and interpretation of data; took part in drafting the article or revising it critically for important intellectual content; agreed to submit to the current journal; gave final approval of the version to be published; and agree to be accountable for all aspects of the work.

## Notes on contributors

***Jonas Lind***, M.D., Ph.D. is a Consultant Neurologist at the Department of Neurology, Internal Medicine, County Hospital Ryhov, Jönköping and an Adjunct Senior Lecturer at the Division of Neurobiology, Department of Biomedical and Clinical Sciences, Linköping University, Linköping, Sweden.

***Niclas Stensson***, M.Sc., Ph.D is affiliated with the Pain and Rehabilitation Centre, and Department of Health, Medicine and Caring Sciences, Linköping University, Linköping, Sweden.

***Björn Gerdle***, M.D., Ph.D. is a Professor emeritus and a Consultant at Pain and Rehabilitation Centre, and Department of Health, Medicine and Caring Sciences, Linköping University, Linköping, Sweden.

***Nazdar Ghafouri***, M.D., Ph.D., is a Consultant Physician at Pain and Rehabilitation Centre, and Department of Health, Medicine and Caring Sciences, Linköping University, Linköping, Sweden.

## ORCID

Jonas Lind https://orcid.org/0000-0001-5357-3767

Niclas Stensson https://orcid.org/0000-0001-6081-9673

Björn Gerdle https://orcid.org/0000-0002-4316-1264

Nazdar Ghafouri https://orcid.org/0000-0001-8789-0656
